# The Impact of Aging, Psychotic Symptoms, Medication, and Brain-Derived Neurotrophic Factor on Cognitive Impairment in Japanese Chronic Schizophrenia Patients

**DOI:** 10.3389/fpsyt.2018.00232

**Published:** 2018-05-29

**Authors:** Kiyokazu Atake, Tomoyuki Nakamura, Nobuhisa Ueda, Hikaru Hori, Asuka Katsuki, Reiji Yoshimura

**Affiliations:** ^1^Department of Psychiatry, School of Medicine, University of Occupational and Environmental Health, Kitakyushu, Japan; ^2^Department of Neuropsychiatry, Kurume University, Kurume, Japan; ^3^Tsutsumi Hospital, Okagaki, Japan

**Keywords:** schizophrenia, cognitive impairment, aging, brain-derived neurotrophic factor, Japanese-language version of the Brief Assessment of Cognition in Schizophrenia

## Abstract

**Background:** Cognitive impairment in schizophrenia can result in considerable difficulty in performing functions of daily life or social rehabilitation. Cognitive impairment in schizophrenia is related to various factors, such as the psychotic severity, aging, medication, and brain-derived neurotrophic factor (BDNF). To date, however, no studies investigating the impact of these factors on cognitive functioning in chronic schizophrenia patients have been performed.

**Objective:** The aim of this study is to identify those factors that influence the cognitive functioning in patients with chronic schizophrenia.

**Methods:** Sixty-five of 116 long-term hospitalized chronic schizophrenia patients (63.8 ± 12.1 years old, M/F = 29/36) were enrolled this cross-sectional study. We investigated the relationship among the patients' age, psychotic severity, treatment medication, serum BDNF levels, and cognitive functioning (measured by the Japanese-language version of the Brief Assessment of Cognition in Schizophrenia; BACS-J). Additionally, we performed a multivariable linear regression analysis.

**Results:** According to the partial correlation analysis, certain parameters [i.e., age, chlorpromazine (CP) equivalent, biperiden (BP) equivalent, and serum BDNF] were significantly correlated with cognitive functioning, including working memory (WM), motor function (MF), attention and processing speed (AP), and executive function (EF). For the multivariate analysis, the MF component, which had the highest correlation, was selected as the dependent variable, and the independent variables included age, Manchester Scale for chronic psychosis (ManS) total score, CP equivalent, BP equivalent, serum BDNF, estimated full scale IQ, and years of education. According to the multiple regression analysis of this model, *R* (multiple regression coefficient) was 0.542, the adjusted *R*^2^ (coefficient of determination) was 0.201, and only BP equivalent (β = −0.305, *p* = 0.030), but not age, ManS score, CP equivalent, or serum BDNF, could significantly explain MF at the 5% significant level.

**Conclusion:** In conclusion, aging, medication (administering more antipsychotics or anticholinergics), and serum BDNF concentration are significantly correlated with cognitive dysfunction in chronic schizophrenia patients but not with the severity of psychotic symptoms. Furthermore, only the anticholinergic dosage had a significant causal relationship with MF. Thus, the use of anticholinergics in chronic schizophrenia patients with deteriorating cognitive functioning must be reconsidered.

## Introduction

Cognitive impairments in schizophrenia can result in considerable difficulty in performing functions of daily life or social rehabilitation, and cognitive difficulty persists even after the alleviation of psychotic symptoms. Reintegrating into society is particularly challenging for these patients due to their severe cognitive impairment. Therefore, ascertaining the level of cognitive dysfunction remaining after improvement in acute psychiatric symptoms is important.

Cognitive impairment in schizophrenia is related to various factors, such as psychotic symptoms, aging, medication, and genetic variants. In the current study, we investigate the cognitive functioning in schizophrenia patients using the Japanese language version of the Brief Assessment of Cognition in Schizophrenia (BACS-J). Kaneda et al. (1) investigated the influence of disease and aging on performance on the BACS-J in schizophrenic patients. A multiple regression analysis including all subjects indicated that performance on almost all BACS-J components were attributable to the disease, aging, the level of education, and the duration of illness.

In schizophrenia patients, deterioration in cognitive functioning is a symptom of the disease; additionally, medication also affects functioning. Anticholinergic agents affect cognitive functioning, and antipsychotics, which are used to treat schizophrenia, also cause cognitive function to deteriorate. We previously reported that excessive doses of antipsychotics can cause deterioration in cognitive functioning in chronic schizophrenia patients (2), and switching from polypharmacy to antipsychotic monotherapy improves cognitive functions, such as attention and executive function (EF) (3).

Additionally, serum brain-derived neurotrophic factor (BDNF) levels are positively associated with cognitive functions, such as immediate memory, in chronic schizophrenia patients (4). In addition, Zhang et al. (5) found that low BDNF levels are associated with poor performance on the cognitive factor of the Positive and Negative Syndrome Scale (PANSS) in chronic schizophrenia. In particular, Tumor Necrosis Factor-α (TNF-α) may interact with BDNF and cause cognitive impairment. In contrast, Buchman et al. (6) reported that higher levels of brain BDNF expression are associated with a slower cognitive decline. We previously reported that the serum BDNF levels are positively correlated with verbal memory (VM), attention, and processing speed in chronic schizophrenia (7). Altogether, BDNF is closely associated with symptomatology and cognitive dysfunction in schizophrenia. Indeed, a recent meta-analysis demonstrated that higher levels of BDNF correspond to better performance on several cognitive tests, including reasoning and problem-solving tasks, on the Measurement and Treatment Research to Improve Cognition in Schizophrenia (MATRICS) consensus cognitive battery (MCCB) (8).

Considering these findings, we aim to identify the factor (i.e., psychotic severity, aging, medication, or BDNF) that has the greatest influence on cognitive functioning in patients with chronic schizophrenia. Specifically, the purpose of this study was to determine the factors that are significantly related to cognitive performance in chronic schizophrenia inpatients and those that have the greatest impact on their cognitive impairment. To the best of our knowledge, this study is the first to examine the association between the factors described above and cognitive functioning in long-term hospitalized chronic schizophrenia inpatients.

## Materials and methods

### Subjects

One hundred and sixteen chronic schizophrenia inpatients were recruited from Hiagari Hospital, Tsutsumi Hospital, Shin-Moji Hospital, and the University of Occupational and Environmental Health. The participants in the present study met the following inclusion criteria: (1) age over 20 years; (2) chronic illness without acute exacerbation; (3) continuous hospitalization for at least 3 years for schizophrenia; and (4) continuous treatment with a stable dose of atypical antipsychotic medication for at least 3 months. The diagnosis of schizophrenia was based on the Structured Clinical Interview for DSM-IV-TR Disorders (SCID) and a comprehensive review of patient medical records. The exclusion criteria were as follows: (1) any comorbid CNS disorder; (2) meeting the DSM-IV-TR criteria for affective, schizoaffective, or schizophreniform psychosis; alcohol or other substance dependence; or mental retardation; (3) receiving antidepressants or mood stabilizers; (4) having received electroconvulsive therapy within the 6 months preceding the study period; and (5) having received clozapine. We used the SCID to screen all participants and excluded those patients with psychiatric disorders. No subjects had a history of neurological, somatic, or psychiatric illnesses. Sixty-five patients (63.8 ± 12.1 years old, M/F = 29/36) who met the above-mentioned criteria, completed all assessments, including blood extraction, and provided informed consent were enrolled in the present study. The participants who declined to participate or did not participate for another reason were not disadvantaged in any treatment modalities due to their lack of participation in this study.

### Clinical assessment

The Global Assessment of Functioning (GAF) was used to assess the general functioning of the participants. The participants' psychotic symptoms were assessed by their primary doctors using the Manchester Scale for chronic psychosis (ManS). Their cognitive functioning was assessed using the BACS-J. Adverse effects, such as extrapyramidal symptoms, were measured using the Drug-Induced Extrapyramidal Symptoms Scale (DIEPSS). The use of antipsychotic and anticholinergic drugs was considered in terms of chlorpromazine (CP) and biperiden (BP) equivalents.

### Neurocognitive functioning test

Cognitive functioning was assessed by trained psychiatrists using the BACS-J. The BACS is an instrument to evaluate the cognitive functioning in schizophrenic patients. This assessment consists of six tests, including VM, working memory (WM), motor function (MF), attention and processing speed (AP), EF, and composite scores (CS) (9). Patients with chronic schizophrenia have severe impairments that range from one and a half to two standard deviations below healthy control subjects in VM, WM, motor speed, attention, EFs, and verbal fluency (9). The BACS-J has established reliability and validity and is designed to measure cognitive functioning in schizophrenia patients. The primary measures of each subtest of the BACS-J were standardized by creating z-scores (i.e., the mean of the healthy controls was set to zero, and the standard deviation was set to one). All data for the healthy controls were obtained from a study conducted by Kaneda et al., and a composite score was calculated by averaging all z-scores for the six primary measures (10). The influence of age was adjusted using age-matched cohorts of controls to calculate the BACS-J z-scores for each schizophrenia patient in the present study.

### Intelligence test

The IQs of the participants were estimated using the Japanese Adult Reading Test: JART (11, 12), which is a Japanese version of the National Adult Reading Test, to exclude mental retardation and estimate the relevance of IQ for cognitive functioning. This test reflects the premorbid IQs in Japanese patients with schizophrenia (13).

### BDNF measurement

All blood samples were obtained between 7:00 and 10:00 a.m. after fasting on the day the clinical assessment was performed. Fifteen milliliters of venous blood were drawn from subjects in the supine position after the subjects rested in the supine position overnight. The serum samples were quickly separated in a centrifuge and stored at −80°C until assay. The serum levels of BDNF were assayed by ELISA using a Milliplex MAP Kit (HNDG3MAG-36K) on a Milliplex Analyzer 4.2 MAGPIX machine (Millipore) according to the manufacturer's instructions.

### Statistical analysis

For the statistical analysis, we supposed that the present studies' sample is based on the normal distribution same as the Kaneda's previous study. Pearson's correlation coefficients were examined to identify the correlations between the patients' clinical variables and serum BDNF levels and scores on each BACS-J neuropsychological test component. Subsequently, we performed a partial correlation analysis to examine the correcter relationship among the parameters without influencers. In the analysis of the partial correlations between the BACS-J z-scores and the clinical parameters, the six task scores and the composite score on the BACS-J were set as the dependent variables, while the clinical parameters, including medication information and clinical conditions, and the serum BDNF concentration, were set as the independent variables. Parameters thought to influence the results of the test battery, such as estimated full-scale IQ and years of education, were set as control variables. Furthermore, we performed a regression analysis to investigate the causality. In the multivariable linear regression analysis, we analyzed the linearity of the BACS-J components that were statistically significant in the partial correlation analysis and set the parameters of interest in the current study (i.e., age, CP equivalent, BP equivalent, and serum BDNF) as independent variables. The results were considered significant at *p* < 0.05. All statistical analyses were performed using SPSS software (SPSS version 23.0J; SPSS, Tokyo, Japan).

### Consent

This study protocol was approved by the review board of our institute; the Ethics Committee of the University of Occupational and Environmental Health. Written informed consent was obtained from all subjects who participated in this study in accordance with the Declaration of Helsinki.

## Results

### Background data of participants

Sixty-five of 116 subjects (63.8 ± 12.1 years old, M/F = 29/36) who consented to participate in the study and provided full data for all assessment items were enrolled in the study. On the occasion of statistical analysis, we supposed that these samples are based on the normal distribution in the track of the Kaneda's previous study. Across all subjects (*n* = 65), the mean number of administered antipsychotic agents was 2.0 ± 1.1. The CP equivalent dose of the antipsychotic agents was 908.3 ± 627.1 mg/day, and the BP equivalent dose of the anticholinergic drugs was 2.01 ± 2.58 mg/day. The mean GAF score was 38.2 ± 15.0, and the mean ManS total score was 12.6 ± 5.8; thus, the disease severity of the enrolled patients ranged from mildly to moderately ill. The mean education level was 11.4 ± 2.1 years, and the premorbid IQ, which was calculated according to the JART, was 90.8 ± 11.1. The demographic data of the enrolled participants are shown in Table 1. Additionally, the mean BACS-J z-score of six components in the present study were as follows, respectively, i.e., VM, −2.79 ± 1.33; WM, −2.12 ± 1.39; MF, −2.63 ± 1.86; VF, −1.60 ± 1.08; AP, −2.54 ± 1.37; EF, −2.64 ± 2.12; CS, −2.10 ± 0.99.

**Table 1:** Demographic data of the participants.

Sex (male/female)	29/36
Age (years)	63.8 ± 12.1
Number of antipsychotics	2.00 ± 1.05
CP equivalent (mg/day)	908.3 ± 627.1
BP equivalent (mg/day)	2.01 ± 2.58
ManS total score	12.6 ± 5.8
DIEPSS total score	4.35 ± 3.78
GAF score	38.2 ± 15.0
Estimated full scale IQ	90.8 ± 11.1
Years of education	11.4 ± 2.1

*Values represent the means ± standard deviations. CP, chlorpromazine; BP, biperiden; ManS, Manchester Scale for chronic psychosis; DIEPSS, Drug-Induced Extrapyramidal Symptoms Scale; GAF, Global Assessment of Functioning*.

### Simple correlation analyses

Significant correlations were observed between (1) working memory (WM) and age (*r* = −0.310, *p* = 0.012); (2) attention and processing speed (AP) (*r* = −0.331, *p* = 0.007) and executive function (EF) (*r* = −0.316, *p* = 0.010) and CP equivalent dosage; (3) verbal memory (VM) (*r* = −0.270, *p* = 0.029), motor function (MF) (*r* = −0.403, *p* = 0.001), AP (*r* = −0.262, *p* = 0.035), composite scores (CS) (*r* = −0.255, *p* = 0.041), and BP equivalent dosage; (4) AP (*r* = 0.340, *p* = 0.006), EF (*r* = 0.314, *p* = 0.011), and the estimated full-scale IQ; (5) VM (*r* = 0.258, *p* = 0.045), AP (*r* = 0.261, *p* = 0.042), CS (*r* = 0.261, *p* = 0.042), and education; and (6) MF (*r* = −0.252, *r* = 0.043) and serum BDNF concentration (Table 2). No correlations were observed between the BACS-J components and the GAF score or DIEPSS total score.

**Table 2:** Analysis of simple correlations between BACS-J z-scores and multiple parameters.

	**VM**		**WM**		**MF**		**VF**		**AP**		**EF**		**CS**	
	***r***	***p***	***r***	***p***	***r***	***p***	***r***	***p***	***r***	***p***	***r***	***p***	***r***	***p***
Age (years)	−0.233^*^	0.061	−0.310^*^	0.012^*^	−0.236^*^	0.058	−0.007	0.956	0.134	0.288	−0.123	0.330	−0.163	0.196
CP equivalent (mg/day)	−0.120	0.341	−0.079	0.531	−0.233^*^	0.062	0.078	0.538	−0.331^*^	0.007^**^	−0.316^*^	0.010^*^	−0.207^*^	0.098
BP equivalent (mg/day)	−0.270^*^	0.029^*^	−0.151	0.229	−0.403^**^	0.001^**^	0.046	0.715	−0.262^*^	0.035^*^	−0.231^*^	0.064	−0.255^*^	0.041^*^
GAF score	0.203^*^	0.105	0.159	0.206	0.132	0.295	0.029	0.821	0.080	0.525	0.090	0.474	0.152	0.228
ManS total score	−0.139	0.271	−0.177	0.159	−0.196	0.117	−0.031	0.809	−0.193	0.123	−0.226^*^	0.070	−0.213^*^	0.088
DIEPSS total score	0.021	0.870	−0.024	0.851	−0.137	0.276	0.064	0.615	−0.106	0.403	−0.096	0.449	−0.058	0.648
Estimated full scale IQ	−0.042	0.741	0.154	0.220	−0.083	0.512	0.213^*^	0.088	0.340^*^	0.006^**^	0.314^*^	0.011^*^	0.213^*^	0.088
Years of education	0.258^*^	0.045^*^	0.158	0.225	0.178	0.169	0.114	0.383	0.261^*^	0.042^*^	0.246^*^	0.056	0.261^*^	0.042^*^
Serum BDNF (ng/ml)	−0.161	0.201	−0.079	0.532	−0.252^*^	0.043^*^	−0.115	0.362	−0.196	0.118	−0.110	0.383	−0.203^*^	0.104

“r” indicates Pearson's product-moment correlation coefficient; *|r| > 0.2, **|r| > 0.4; “p” indicates the p-value; *p < 0.05, **p < 0.01. CP, chlorpromazine; BP, biperiden; GAF, Global Assessment of Functioning; ManS, Manchester Scale for chronic psychosis; DIEPSS, Drug-Induced Extrapyramidal Symptoms Scale; VM, verbal memory; WM, working memory; MF, motor function; VF, verbal fluency; AP, attention and processing speed; EF, executive function; CS, composite score.

### Partial correlation analyses

In the analyses of the partial correlations between the BACS-J z-scores and multiple clinical parameters, the six task scores and the composite score of the BACS-J were set as dependent variables, while the clinical parameters (i.e., CP equivalent, BP equivalent, GAF score, ManS total score, DIEPSS total score, and serum BDNF) were set as independent variables. Additionally, those parameters known to influence the results of the test battery, such as the estimated full-scale IQ and years of education, were set as control variables. By this correction, several significant relationship found in the simple correlation analysis couldn't be confirmed in the partial correlation analysis. The partial correlations were calculated based on the aforementioned conditions. Significant correlations were observed between (1) WM (*r* = −0.297, *p* = 0.022) and age; (2) MF (*r* = −0.290, *p* = 0.026), AP (*r* = −0.336, *p* = 0.009), EF (*r* = −0.310, *p* = 0.017), and CP equivalent dosage; (3) MF (*r* = −0.434, *p* = 0.001) and BP equivalent; and (4) MF (*r* = −0.287, *p* = 0.027) and serum BDNF concentration (Table 3). The BACS-J components were not correlated with the GAF score or DIEPSS total score.

**Table 3:** Analysis of partial correlations between the BACS-J z-scores and multiple parameters.

	**VM**		**WM**		**MF**		**VF**		**AP**		**EF**		**CS**	
	***r***	***p***	***r***	***p***	***r***	***p***	***r***	***p***	***r***	***p***	***r***	***p***	***r***	***p***
Age (years)	−0.053	0.692	−0.297^*^	0.022^*^	−0.144	0.277	−0.087	0.513	0.033	0.803	−0.136	0.305	−0.151	0.255
CP equivalent (mg/day)	−0.183	0.165	−0.042	0.750	−0.290^*^	0.026^*^	0.133	0.317	−0.336^*^	0.009^**^	−0.310^*^	0.017^*^	−0.199	0.132
BP equivalent (mg/day)	−0.231^*^	0.078	−0.025	0.849	−0.434^**^	0.001^**^	0.080	0.545	−0.249^*^	0.057	−0.133	0.315	−0.189	0.152
GAF score	0.121	0.363	0.082	0.537	0.099	0.455	*O*.015	0.910	0.116	0.383	*O*.015	0.908	0.094	0.478
ManS total score	−0.072	0.586	−0.055	0.678	−0.203^*^	0.124	0.058	0.663	−0.168	0.203	−0.088	0.507	−0.105	0.427
DIEPSS total score	0.011	0.937	−0.018	0.890	−0.163	0.218	0.073	0.585	−0.144	0.275	−0.111	0.401	−0.071	0.594
Serum BDNF (ng/ml)	−0.197	0.136	−0.039	0.767	−0.287^*^	0.027^*^	−0.054	0.686	−0.136	0.304	−0.041	0.761	−0.159	0.229

Values were adjusted by including the estimated full-scale IQ and years of education as control variables. “r” indicates the Pearson's product-moment correlation coefficient; *|r| > 0.2, **|r| > 0.4; “p” indicates the p-value; *p < 0.05, **p < 0.01. CP, chlorpromazine; BP, biperiden; GAF, Global Assessment of Functioning; ManS, Manchester Scale for chronic psychosis; DIEPSS, Drug-Induced Extrapyramidal Symptoms Scale; VM, verbal memory; WM, working memory; MF, motor function; VF, verbal fluency; AP, attention and processing speed; EF, executive function; CS, composite score.

### Multiple regression analysis

In the multivariable linear regression analysis, we analyzed the MF component because this component exhibited more significant associations than the other components. We included the parameters of interest in the current study (i.e., age, CP equivalent, BP equivalent, and serum BDNF concentration) as independent variables. Specifically, the MF component was included as the dependent variable, and the independent variables such as age, ManS total score, CP equivalent, BP equivalent, serum BDNF concentration, estimated full-scale IQ, and years of education were loaded all by the forced entry method. Any collinearity was not found among the following independent variables based on the value of variance inflation factor (VIF) (i.e., age, 1.183; ManS total score, 1.209; CP equivalent, 1.483; BP equivalent, 1.411; serum BDNF, 1.074; estimated full-scale IQ, 1.293; educated years, 1.242), respectively. The multiple regression coefficient of this model, R, was 0.542; the adjusted *R*^2^ (coefficient of determination) was 0.201. Additionally, the significance of the *F*-test in the analysis of variance was *p* < 0.007, which was significant at the 1% level. The β-values, which indicate the strength of the effect of each variable, and the significance probability were as follows: age (β = −0.115, *p* = 0.363), ManS total score (β = −0.082, *p* = 0.648), CP equivalent (β = −0.133, *p* = 0.347), BP equivalent (β = −0.305, *p* = 0.030), serum BDNF (β = −0.205, *p* = 0.092), estimated full-scale IQ (β = −0.180, *p* = 0.175), and years of education (β = 0.166, *p* = 0.204) (Table 4). Thus, BP equivalent significantly explained MF at the 5% significance level; in contrast, age, ManS score, CP equivalent, and serum BDNF were not significantly associated with MF. Overall, only the BP equivalent dosage had a causal relationship with MF according to the final analysis.

**Table 4:** Multivariable linear regression analysis of BACS-J motor function and multiple parameters.

	**Motor function**				
	**B**	**SE**	**β**	***t***	***p***
Constant	1.326	2.376		0.558	0.579
Age	−0.019	0.020	−0.115	−0.918	0.363
ManS total Score	−0.027	0.042	−0.082	−0.648	0.520
CP equivalent (mg/day)	0.000	0.000	−0.133	−0.950	0.347
BP equivalent (mg/day)	−0.233	0.104	−0.305	−2.226	0.030^*^
serum BDNF (ng/ml)	−0.057	0.033	−0.205	−1.717	0.092
Estimated full scale IQ	−0.031	0.023	−0.180	−1.374	0.175
Years of education	0.149	0.115	0.166	1.287	0.204

Values were adjusted by including the estimated full-scale IQ and years of education as control variables. “B” indicates the unstandardized coefficient; “SE” indicates the standard error of B; “β” indicates the standardized regression coefficient; and “p” indicates the p-value;

* *p < 0.05. ManS, Manchester Scale for chronic psychosis; CP, chlorpromazine; BP, biperiden*.

## Discussion

Kaneda et al. (1) mentioned that BACS is affected negatively not only by schizophrenia but also by the level of education and aging. However, there were some differences between the Kaneda's paper and the present study. For example, the mean age of participants in the present study was higher (more than 20 years older), and, in addition, the mean z-score of respective components in the present study was lower than in Kaneda et al. (1).

The main finding in the present study was a negative causal relationship between the anticholinergic agent dosage and MF. The partial correlation analyses revealed the following correlations: age and WM (*r* = −0.297, *p* = 0.022); antipsychotic agent dose and MF (*r* = −0.336, *p* = 0.009) or EF (*r* = −0.310, *p* = 0.017); anticholinergic agent dose and MF (*r* = −0.434, *p* = 0.001); and serum BDNF and MF (*r* = −0.287, *p* = 0.027).

Many studies have compared BACS scores before and after the administration of antipsychotic medication (14, 15). Additionally, we previously reported an association between cognitive functioning and schizophrenia medication use in which polypharmacy adversely affected cognitive functioning (2), and switching to antipsychotic monotherapy from polypharmacy improved cognitive functions, including AP (3). The results of the current study are similar to those of these reports.

To date, multiple studies have reported that anticholinergic drugs may be associated with cognitive impairment, particularly in older adults (16–18). However, few studies have focused on MF in investigations of the anticholinergic influences on cognitive impairment. The MF component of the BACS-J assesses the agility and accuracy of hand movements as patients place a token in a container. The results may be affected by the degree of extrapyramidal symptoms independently of the anticholinergic dosage even though MF and the DIEPSS total score were not correlated in this study. Therefore, the effect of anticholinergics on MF is considered independent of extrapyramidal symptoms.

Blood BDNF concentrations reflect brain BDNF levels in rats, mice, or pigs (19), so peripheral BDNF levels might partially reflect a synthesis, secretion and metabolism of brain BDNF in schizophrenia patients. In short, measuring blood BDNF levels may be valuable for assuming brain BDNF dynamics. A meta-analysis showed that the blood BDNF concentration in schizophrenia patients is lower than that in healthy controls (20, 21). BDNF levels are related to the negative symptoms (22). Chiou et al. (23) also reported that pretreatment negative symptoms played a pivotal role in the trajectories of the serum BDNF levels. Thus, BDNF likely affects cognitive functioning via the negative symptoms. We also reported that decision making, which was tested using the Iowa Gambling Task, was influenced by the PANSS-G scores and serum BDNF levels in chronic schizophrenia patients (24); however, in the present study, we found a significant relationship between MF and serum BDNF in chronic schizophrenia patients. Moreover, these effects were detected not only in the simple correlation analysis but also in the partial correlation analysis, which was adjusted for the estimated full-scale IQ and years of education. Thus, BDNF likely influences certain cognitive functions, such as decision making or MF, in chronic schizophrenia patients.

From an anatomical perspective, Rao et al. (25) reported that BDNF protein levels in the prefrontal cortex gray matter were significantly lower in elderly patients in both non-psychiatric and psychiatric patients, while BDNF levels in the white matter did not significantly decrease with age in either group. In animal aging studies, older age is associated with reduced BDNF expression in the prefrontal cortex and hippocampus (26). Therefore, BDNF may be considered a useful biomarker for re-examining the assumed neurodegenerative course in schizophrenia. The prefrontal cortex BDNF levels linearly decrease from 20 to 80 years of age in non-psychiatric samples. In schizophrenia, the age effect is similarly linear in younger patients, but a decline does not occur in older patients. Thus, the prefrontal cortex BDNF levels do not follow a normative linear age effect in schizophrenia patients with increasing age, which may represent a “floor effect” due to an earlier decline (25). In summary, aging in schizophrenia patients could lessen the influence of the disease, and BDNF changes may decelerate as schizophrenia patients' age. These hypotheses could explain the correlation between cognitive functioning (MF) and age observed in the present study.

Many previous reports using small sample sizes (at least a few dozen) have demonstrated a positive correlation between peripheral BDNF levels and cognition as follows: higher levels of BDNF are associated with better cognitive functioning in schizophrenia. However, this finding is not robust. In the present study, we demonstrated a negative correlation between serum BDNF and MF in chronic schizophrenia patients. Thus, whether peripheral BDNF reflects cognitive functioning in schizophrenia patients remains unknown. According to a recent report, peripheral BDNF concentrations are significantly lower in schizophrenia patients than those in healthy subjects. In addition, BDNF is not correlated with the severity of the positive and negative symptom (20) and cognitive impairment (27). Considering these evidences, we may be able to think as follows. Low BDNF may contribute to the pathogenesis of schizophrenia indeed, however, it may not contribute to its cognitive impairments directly. We cannot explain the cause of the negative correlation between MF and serum BDNF.

Regarding the relationship between BDNF and motor functioning, many articles focus on Parkinson's disease, where limited MF appears to indicate lower levels of peripheral BDNF. The findings in the present study, i.e., that the anticholinergic dosage is strongly negatively correlated with MF, depend on various confounding factors, such as the effects of schizophrenia, other medications, and other factors affecting the participants' cognitive states. The cause of this discrepancy is currently unknown.

As described above, the multiple regression analysis considered seven parameters (i.e., age, ManS total score, CP equivalent, BP equivalent, serum BDNF, estimated full-scale IQ, and years education) as independent variables. The standardized regression coefficient “β” suggests that BP equivalent dosage had the greatest influence on cognitive impairment, followed in order by serum BDNF concentration, estimated full-scale IQ, years of education, CP equivalent dosage, age, and ManS total score. However, only the BP equivalent dosage (β = −0.305, *p* = 0.030) significantly explained MF at the 5% significance level. Thus, treating patients with lower doses of anticholinergic drugs is optimal for improving MF. We may wish to reconsider the prescriptions given to patients who have been hospitalized for a long time to determine whether we can reduce their intake of anticholinergic agents.

Our study has several limitations. First, this study was a cross-sectional survey without control subjects. Although we considered the factor of age, the data do not directly indicate the effect of aging. Second, the sample size was too small to characterize the relationships described above. Third, the study considered the effect of antipsychotic and anticholinergic drugs but did not consider other drugs, such as benzodiazepine compounds. Fourth, the data were not adjusted for other control variables, such as smoking, obesity, platelet count, lifestyle factors, sleep, and diet. Fifth, the MF might be influenced by age and medication and the impairment of MF might affect other components of cognitive function, however the present study did not consider the points. Finally, we did not analyze of possible differences in any of these outcomes between male and female patients. Further studies considering these variables should be performed to confirm the results of this preliminary study.

## Conclusions

In summary, we confirmed that aging, medication (administering more antipsychotic or anticholinergic drugs) and higher serum BDNF concentrations were significantly correlated with cognitive dysfunction in chronic schizophrenia but not the severity of psychotic symptoms. Additionally, the BP equivalent dosage had the highest impact on MF. Diminishing the use of anticholinergic drugs may improve cognitive dysfunction, particularly in terms of motor functioning, in chronic schizophrenia patients.

## Author contributions

KA and RY conceived and designed the experiments. KA, TN, and NU performed the experiments. KA, HH, and AK analyzed the data. KA prepared the manuscript. RY edited the manuscript.

### Conflict of interest statement

The authors declare that the research was conducted in the absence of any commercial or financial relationships that could be construed as a potential conflict of interest.
